# Differential vulnerability of interneurons in the epileptic hippocampus

**DOI:** 10.3389/fncel.2013.00167

**Published:** 2013-10-01

**Authors:** Markus Marx, Carola A. Haas, Ute Häussler

**Affiliations:** ^1^Experimental Epilepsy Research, Department of Neurosurgery, University of FreiburgFreiburg, Germany; ^2^Bernstein Center Freiburg, University of FreiburgFreiburg, Germany; ^3^BrainLinks-BrainTools, Cluster of Excellence, University of FreiburgFreiburg, Germany

**Keywords:** kainate injection, glutamic acid decarboxylase, parvalbumin, neuropeptide Y, temporal lobe epilepsy, septotemporal axis

## Abstract

The loss of hippocampal interneurons has been considered as one reason for the onset of temporal lobe epilepsy (TLE) by shifting the excitation-inhibition balance. Yet, there are many different interneuron types which show differential vulnerability in the context of an epileptogenic insult. We used the intrahippocampal kainate (KA) mouse model for TLE in which a focal, unilateral KA injection induces *status epilepticus* (SE) followed by development of granule cell dispersion (GCD) and hippocampal sclerosis surrounding the injection site but not in the intermediate and temporal hippocampus. In this study, we characterized the loss of interneurons with respect to septotemporal position and to differential vulnerability of interneuron populations. To this end, we performed intrahippocampal recordings of the initial SE, *in situ* hybridization for glutamic acid decarboxylase 67 (GAD67) mRNA and immunohistochemistry for parvalbumin (PV) and neuropeptide Y (NPY) in the early phase of epileptogenesis at 2 days and at 21 days after KA injection, when recurrent epileptic activity and GCD have fully developed. We show that SE extended along the entire septotemporal axis of both hippocampi, but was stronger at distant sites than at the injection site. There was an almost complete loss of interneurons surrounding the injection site and expanding to the intermediate hippocampus already at 2 days but increasing until 21 days after KA. Furthermore, we observed differential vulnerability of PV- and NPY-expressing cells: while the latter were lost at the injection site but preserved at intermediate sites, PV-expressing cells were gone even at sites more temporal than GCD. In addition, we found upregulation of GAD67 mRNA expression in dispersed granule cells and of NPY staining in ipsilateral granule cells and ipsi- and contralateral mossy fibers. Our data thus indicate differential survival capacity of interneurons in the epileptic hippocampus and compensatory plasticity mechanisms depending on the hippocampal position.

## INTRODUCTION

Interneurons play a crucial role in balancing neuronal activity in the brain. In epilepsy the loss of inhibitory interneurons has been associated with the emergence of epileptic seizures (for review, see Lopes da Silva et al., 1994; Olsen and Avoli, 1997; Fritschy, 2008). In temporal lobe epilepsy (TLE) interneurons show differential vulnerability to an epileptogenic insult (Bouilleret et al., 2000a,b; Magloczky and Freund, 2005; Kuruba et al., 2011) characteristic for their particular expression pattern of interneuron markers: interneurons commonly express glutamic acid decarboxylase (GAD), the key enzyme for synthesis of the inhibitory transmitter gamma-aminobutyric acid (GABA; Ribak et al., 1978; Freund and Buzsaki, 1996), but they can be differentiated according to the selective expression of calcium-binding proteins [calbindin, parvalbumin (PV), calretinin] and/or co-transmitters [e.g., somatostatin, neuropeptide Y (NPY), cholecystokinin] (Freund and Buzsaki, 1996).

Among the most vulnerable interneurons in human TLE and in animal models for TLE are those expressing PV (Bouilleret et al., 2000a; Kuruba et al., 2011) which comprise basket cells, axo-axonic cells and bistratified cells (Freund and Buzsaki, 1996; Somogyi and Klausberger, 2005). However, there are controversial reports in the literature ranging from substantial loss of PV-expressing cells (Bouilleret et al., 2000a), to a transient loss of PV expression (Sloviter et al., 1991; Wittner et al., 2001) or only marginal reduction of PV-expressing cells (Wyeth et al., 2010), depending on the hippocampal region, the degree of hippocampal sclerosis and the particular patient or animal model.

Another interneuron population which is partially lost in TLE expresses the co-transmitter NPY (Sloviter and Lowenstein, 1992; Sperk et al., 1992; Kuruba et al., 2011). In the healthy hippocampus the majority of these cells is positioned in the hilus (Deller and Leranth, 1990; Sperk et al., 2007) and in *stratum oriens* of CA3 and CA1 (Freund and Buzsaki, 1996). In addition to the loss of NPY-expressing cells in TLE, the upregulation of NPY in granule cells and mossy fibers has been described in several animal models (Sperk et al., 1992; Makiura et al., 1999; Vezzani et al., 1999; Arabadzisz et al., 2005), most likely reflecting a compensatory mechanism.

Vulnerability of interneurons has also been described in the intrahippocampal kainate (KA) mouse model for TLE, which shows histological changes closely resembling human TLE including hippocampal sclerosis and granule cell dispersion (GCD; Suzuki et al., 1995; Bouilleret et al., 1999; Riban et al., 2002). Most investigations in this model focused on the septal hippocampus (close to the injection site) as it shows prominent cell loss and strong GCD, however, our previous study revealed that *status epilepticus* (SE) and recurrent epileptiform activity involve the whole septotemporal extent of both hippocampi (Häussler et al., 2012). This indicates that structural changes in the network beyond the septal hippocampus are highly likely. Indeed, we described that the intermediate hippocampus constitutes a transition zone where GCD reduces to normal width, but where neurogenesis, which is lost in the septal hippocampus (Heinrich et al., 2006), is even increased compared to controls (Häussler et al., 2012). It is, however, still unknown whether and how the interneuron network is affected in this area – this might be crucial for network balance and seizure generation.

To address this question, we investigated whether there is a location-dependent vulnerability of interneurons along the whole septotemporal axis of both hippocampi early and late after KA injection. We present quantitative data showing a substantial loss of GAD-expressing interneurons beyond the area where GCD occurred, with differential vulnerability of PV- and NPY-expressing interneurons. In addition, NPY was upregulated in granule cells and mossy fibers in a time- and position-dependent manner most likely reflecting particular compensatory mechanisms at different septotemporal sites.

## MATERIALS AND METHODS

### ANIMALS

Experiments were carried out with adult (8–10 weeks) male C57Bl/6N mice (Charles River, Sulzfeld, Germany). Mice were kept in a 12 h light/dark cycle at room temperature (RT; 22 ± 1°C) with food and water *ad libitum*. All animal procedures were carried out in accordance with the guidelines of the European Community’s Council Directive of 22 September 2010 (2010/63/EU) and approved by the regional council.

### KAINATE INJECTION AND ELECTRODE IMPLANTATION

Unilateral, intrahippocampal KA injections were performed as previously described (Heinrich et al., 2006; Häussler et al., 2012). In brief, mice were anesthetized (100 mg/kg ketamine hydrochloride, 5 mg/kg xylazine, and 0.1 mg/kg atropine; i.p.) and placed into a stereotaxic frame (David Kopf Instruments, Tujunga, CA, USA) in flat skull position. 50 nl (1 nmol) of a 20 mM KA solution (Tocris, Bristol, UK) in 0.9% sterile NaCl solution were injected into the right dorsal hippocampus within 1 min [coordinates from bregma (in mm): anterio-posterior (AP) = -2.0, medio-lateral (ML) = -1.4, dorso-ventral (DV) = -1.9]. After injection, some mice were immediately implanted with custom-made platinum-iridium wire electrodes (ø = 125 μm, Teflon insulated, World Precision Instruments, Sarasota, FL, USA) at four positions along the septotemporal axis of the ipsilateral and at one position in the contralateral hippocampus [coordinates (in mm): (1) AP = -2.0, ML = -1.4, DV = -1.9; (2) AP = -2.8, ML = -2.0, DV = -2.0; (3) AP = -3.4, ML = -2.75, DV = -2.75, (4) AP = -3.8, ML = -2.5, DV = -4.0, (contra) AP = -2.0, ML = +1.4, DV = -1.9]. Stainless steel jeweler’s screws positioned in the skull above the prefrontal cortex served as ground and reference. Electrodes were fixed to the skull with cyanoacrylate and dental cement and soldered to a connector which was permanently mounted on the skull with dental cement. After recovery from anesthesia, mice with implanted electrodes were recorded for several hours to monitor SE. Mice, destined for histological analyses were not implanted with electrodes to ensure better preservation of the tissue, but it was ensured that behavioral manifestation of SE was comparable to the recorded group.

### *IN VIVO* INTRAHIPPOCAMPAL RECORDINGS

For recordings of hippocampal local field potential (LFP) activity *in vivo*, mice were connected to a miniature preamplifier [Multi Channel Systems (MCS), Reutlingen, Germany]. Signals were amplified (1000-fold, band-pass 1 Hz–5 kHz; MCS) and digitized (sampling rate 10 kHz, Power1401 analog-to-digital (A/D) converter, Spike2 software, Cambridge Electronic Design, Cambridge, UK). Animals were recorded during the initial SE and at 3 and 21 days after KA injection to ensure that they developed chronic TLE. Electrode positions were verified in Nissl-stained sections as described previously (Häussler et al., 2012).

### PERFUSION AND TISSUE PREPARATION

Mice were deeply anesthetized at 2 and 21 days after KA injection and transcardially perfused with 0.9% NaCl solution followed by paraformaldehyde [PFA, 4% in 0.1 M phosphate buffer (PB), pH 7.4]. The brains were post-fixed in the same fixative for 4 h at 4°C, and either cryoprotected (20% sucrose, overnight, 4°C), frozen in isopentane and sectioned with a cryostat (50 μm, coronal plane) or transferred into PB and cut on a vibratome (Leica, VT1000S, Bensheim, Germany; 50 μm, coronal plane).

### IMMUNOCYTOCHEMISTRY

For immunocytochemistry, sections were processed using a free-floating procedure. They were preincubated in 0.25% Triton X-100 and 10% normal serum in PB for 30 min and incubated with the primary antibody (4 h at RT + overnight at 4°C). The following primary antibodies were used: rabbit anti-PV (1:2500, Swant, Bellinzona, Switzerland), rabbit anti-NPY (1:3000, Abcam, Cambridge, UK), rabbit anti-GAD 65/67 (GAD65/67, 1:4000, Millipore, Temecula, CA, USA). Secondary antibodies were coupled to Cy^TM^2 (1:200) or Cy^TM^3 (1:400, Jackson ImmunoResearch Laboratories, West Grove, PA, USA) and counterstaining was performed with 4′,6-diamidino-2-phenylindole (DAPI, 1:10000) in the dark for 2–3 h at RT. Sections were coverslipped with anti-fading mounting medium (IMMU-Mount, ThermoShandon, Dreieich, Germany).

### *IN SITU* HYBRIDIZATION

Glutamic acid decarboxylase 67 mRNA was localized by *in situ* hybridization (ISH) with digoxigenin (DIG)-labeled cRNA probes generated by *in vitro* transcription as described earlier (Kulik et al., 2003). Cryostat sections were pretreated in hybridization buffer (50% formamide, 4× SSC (1× SSC = 0.15 M NaCl, 0.015 M sodium citrate, pH 7.0), 50 mM NaH_2_PO_4_, 250 μg/ml heat-denatured salmon sperm DNA, 100 μg/ml tRNA, 5% dextransulfate and 1% Denhardt’s solution) diluted with 2× SSC (1:1) for 15 min and prehybridized in hybridization buffer for 60 min at 45°C. Hybridization was performed in the same buffer including DIG-labeled GAD67 anti-sense or sense cRNA probes (50 ng/ml) at 45°C overnight. After hybridization, the brain sections were washed in 2× SSC (2 × 15 min, RT), 2× SSC and 50% formamide (15 min, 55°C), 0.1× SSC and 50% formamide (15 min at 55°C), 0.1× SSC (2 × 15 min, 55°C) and finally in Tris-buffered saline (TBS, 2 × 10 min, RT). Blocking was performed in blocking buffer (1% blocking reagent in TBS, 60 min, RT). Immunological detection of DIG-labeled hybrids was performed with an anti-DIG antibody conjugated with alkaline phosphatase (1:1500, raised in sheep, Roche, Mannheim, Germany) following standard protocols. Sections were coverslipped in Kaiser’s glycerol gelatine.

### FLUORO-JADE B STAINING

The success of KA injections was monitored by Nissl (data not shown) or Fluoro-Jade B stainings to monitor cell death. Sections were mounted on gelatine-coated microscope slides, air-dried and transferred into 0.06% potassium permanganate solution (15 min), followed by 0.0004% Fluoro-Jade B solution (30 min). Sections were cleared in xylene and coverslipped with Hypermount.

### MICROSCOPY AND COUNTING PROCEDURES

Histological sections were analyzed with a microscope equipped with appropriate fluorescence filters (Axioplan 2, Zeiss, Göttingen, Germany), photomicrographs were taken with a digital camera and processed with Axiovision software (Zeiss). Identical exposure times were used for sections that were compared. ISH sections were analyzed using brightfield microscopy with the same equipment. In the photomicrographs of ISH sections, GAD67 mRNA-positive cells were counted using ImageJ software (National Institutes of Health, Bethesda, Maryland, USA) with the integrated Cell Counter plugin. For quantifications, representative coronal sections were selected at four positions along the septotemporal axis according to the following positions given in the Allen Mouse Brain Atlas (©2012 Allen Institute for Brain Science: ): AP relative to bregma (in mm); (1): -1.35, (2): -2.15, (3): -2.98, (4): -3.55. As regions of interest (ROI) we marked the hilus or the complete hippocampus in each section, respectively. Only mice for which sections at each of these positions from the ipsilateral and contralateral hippocampus were available were used for quantification. To compare densities of GAD67 mRNA-positive cells in epileptic and control mice at these particular positions, all GAD67 mRNA-positive interneurons within each ROI were manually marked, counted and the density was calculated using the area of the ROI (values are given in cells/mm^2^). The densities were then averaged across animals for each position. The same method was applied to quantify PV-positive interneurons in immunocytochemically stained sections.

For densitometric analysis of Fluoro-Jade B-positive cells images with equal illumination were taken and the same threshold was applied for every image. The total area of the hippocampus was divided by the area of pixels with brightness exceeding threshold resulting in the relative area of positive pixels as a measure for the amount of cell death.

### DATA ANALYSIS

For visualization of different densities of GAD67 mRNA-positive cells in all hippocampal areas, a custom-made C# program (Microsoft^®^ Visual Studio Professional 2010, Redmond, WA, USA) was developed to create heatmap images in false-color along the septotemporal axis. Images from representative ISH-stained sections were selected for each of the four positions for controls and 2 and 21 days after KA. Using the GAD67 mRNA-positive cells marked in ImageJ, where the position of each marked cell defines an x- and y-coordinate in a coordinate system, a basic matrix was placed centrally onto the position of each cell. The matrix consisted of radially decreasing intensity values (radius 100 pixels in the original image, values linearly decreasing from 100 in the center to 0 in the periphery). Overlapping basic matrix values of different cells were summed resulting in high intensity values where cells were located close to each other. To allow comparison, the values were normalized to the maximal intensity value for each septotemporal position. Finally, the matrices were transferred into grayscale (i.e., scaled to values between 0 and 255) and allocated to a new RGB color space (gray indicates no cells, red indicates highest cell density).

### STATISTICAL ANALYSIS

For all values, mean and standard error of the mean (SEM) are given. Statistical comparison was made with a one-way analysis of variance (ANOVA) followed by Tukey’s multiple comparison test. Significance thresholds were as follows: *p < 0.05, **p < 0.01, ***p < 0.001. Statistical analysis was performed with GraphPad Prism version 5.01 (GraphPad Software, San Diego, CA, USA).

## RESULTS

### STATUS EPILEPTICUS IS FOLLOWED BY EXTENSIVE CELL DEATH

**FIGURE 1:**
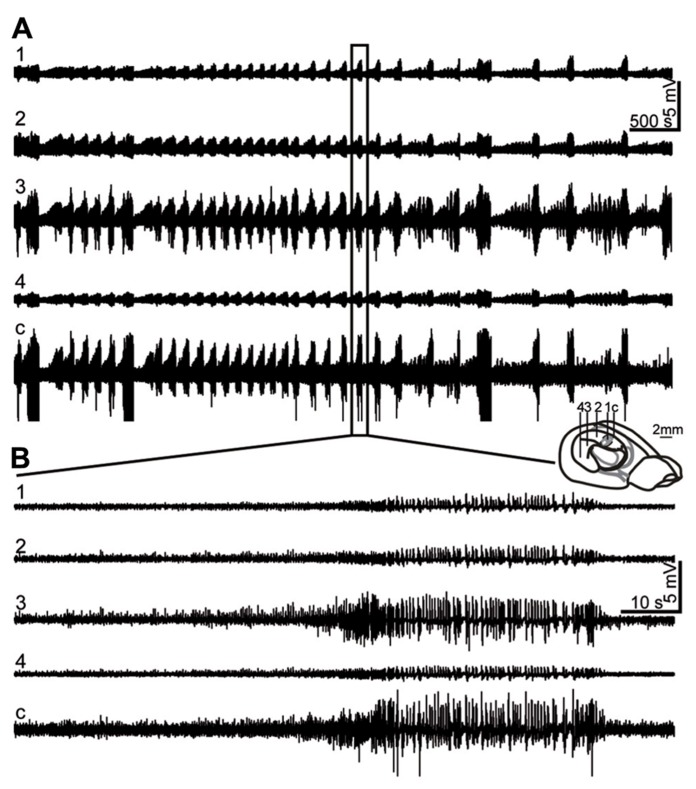
**Representative LFP recording during status epilepticus (SE).** **(A, B)** LFPs were recorded at 4 positions (1: most septal, 4: most temporal) in the ipsilateral and at one position in the contralateral (c) hippocampus (for illustration of electrode positions see schematic brain drawing) starting from ~2 h after awakening from surgery. SE was characterized by a repetitive pattern of spike-and-wave discharges with short intermittent depression periods, accompanied by behavioral signs such as convulsive movements, rotation or immobility. **(B)** Enlargement of **(A)**. SE activity was strongest in the intermediate and contralateral hippocampus at this time point.

To monitor SE, a series of mice was implanted with hippocampal electrodes and LFPs were recorded for 2–3 h after mice had awakened from anesthesia. SE activity, consisting of a repetitive pattern of high amplitude population spikes and spike-and-wave discharges with intermittent short depression periods, was recorded at all septotemporal positions of the ipsilateral hippocampus and in the contralateral hippocampus. At the time point of recording, amplitudes were largest in the ipsilateral intermediate and the contralateral hippocampus in all mice (*n* = 6, **Figures 1A,B**). This is in accordance with our previous study (Häussler et al., 2012), where quantification revealed a significant increase in power in the theta band (3–8 Hz) in the intermediate, temporal and contralateral hippocampus during SE. Simultaneous with pathophysiological activity, mice showed behavioral SE, including rotations, chewing or convulsive movements of the forelimbs, alternating with immobility. This pattern continuously persisted for 6–10 h after KA injection and subsided spontaneously.

**FIGURE 2:**
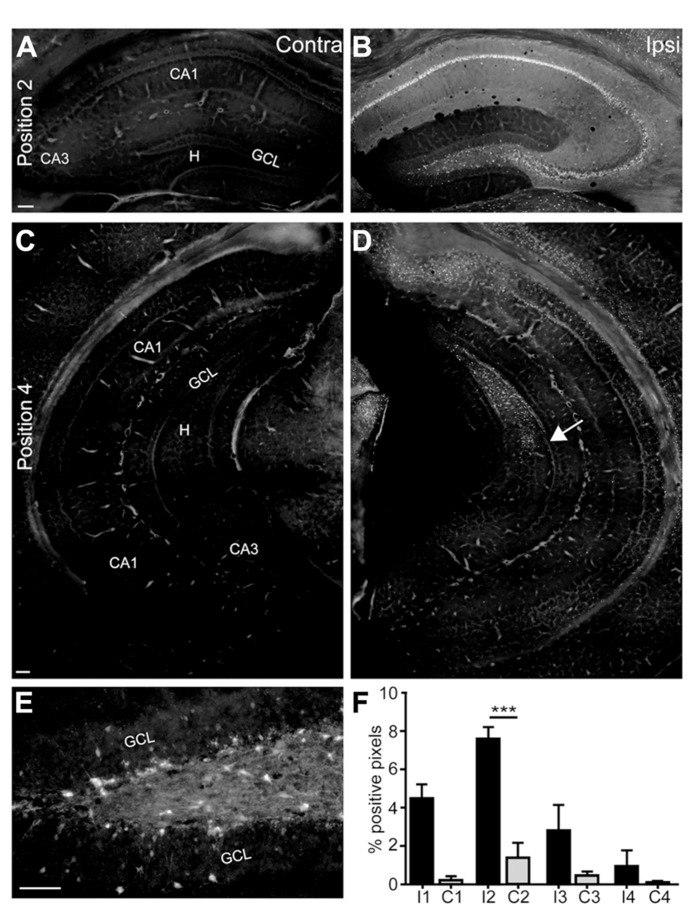
**Status epilepticus is followed by prominent neuronal death in the septal ipsilateral hippocampus.** **(A–E)** Representative Fluoro-Jade B-stained sections of a KA-injected mouse at one day after KA injection to monitor neuronal degeneration. **(A, C)** The contralateral hippocampus was devoid of any cell death in the septal **(A)** and intermediate and temporal hippocampus **(C)**. **(B)** In the ipsilateral hippocampus prominent Fluoro-Jade B staining occurred in the pyramidal cell layer of CA3 and CA1 and in the hilus, but single Fluoro-Jade B-positive cells were also located in *strata oriens* and *radiatum *of CA3 and CA1 and in the inner and outer portion of the granule cell layer. An enlargement of the hilus and granule cell layer, displaying the shape of Fluoro-Jade B-positive cells is shown in **(E)**. **(D)** Fluoro-Jade B-positive cells were also visible in the intermediate hippocampus but the temporal hippocampus was devoid of dying cells. The arrow marks the most temporally located Fluoro-Jade B-positive cell group. **(F)** Densitometric analysis of Fluoro-Jade B-positive areas relative to the area of the whole hippocampus in the same section at four positions along the septotemporal axis (see scheme in **Figure 1**) in the ipsilateral and contralateral hippocampus. Values are displayed as mean ± SEM. A significant difference was observed at position 2 (*n* = 5, *p* < 0.001, ANOVA, ***p < 0.001 Tukey’s post-test). Scale bars: **A–D**, 100 μm; **E**, 50 μm. GCL, granule cell layer; H, hilus; CA1, CA2, CA3, *cornu ammonis*; I1, ipsilateral position 1; C1, contralateral position 1.

One day after KA injection prominent cell death occurred mainly close to the injection site in CA1, CA3 and the hilus of the ipsilateral hippocampus, as shown by Fluoro-Jade B stainings (*n* = 5, **Figures 2B,D,E**). The contralateral hippocampus was devoid of cell loss (**Figures 2A,C**) or a group of dying cells was observed in distal CA1 (3/5 mice). Ipsilateral cell loss following focal KA injection extended to the intermediate hippocampus (**Figure 2D**) but the temporal hippocampus was unaffected. Densitometric analysis revealed prominent differences between cell loss in the ipsilateral and contralateral hippocampus, which, however, only were significant at position 2 (**Figure 2F**; ANOVA: *p* < 0.001, Tukey’s post-test: *p* < 0.001), most likely due to variable extent of cell death mainly in CA1 across mice. In addition to strong cell loss in the pyramidal cell layer in CA3 and CA1 and hilar mossy cells, Fluoro-Jade B-positive cells were also positioned in *strata radiatum* and *oriens* of CA3 and CA1 (**Figure 2B**) and in the inner and outer portion of the dentate granule cell layer (**Figure 2E**). These positions are usually associated with interneurons.

### POSITION-DEPENDENT LOSS OF GAD67 mRNA-EXPRESSING INTERNEURONS IN THE EPILEPTIC MOUSE HIPPOCAMPUS

Glutamic acid decarboxylase 67 is expressed in nearly all GABAergic interneurons (Freund and Buzsaki, 1996). To monitor the distribution of hippocampal interneurons along the septotemporal axis after focal KA injection into the septal hippocampus, we performed ISH for GAD67 mRNA expression at 2 and 21 days after injection.

**FIGURE 3:**
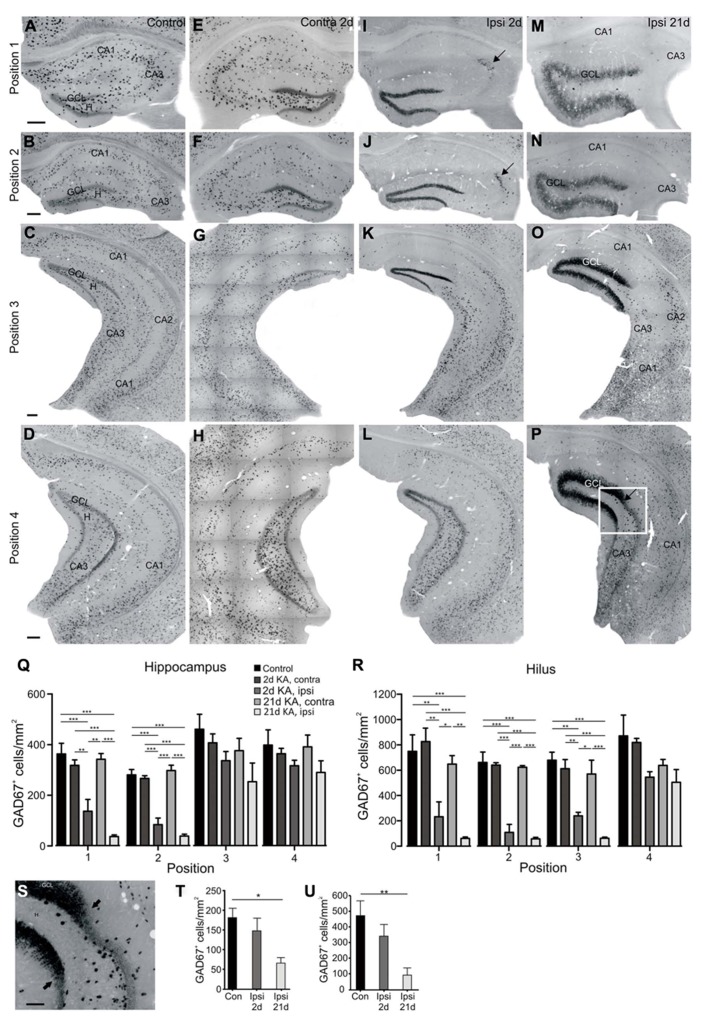
**Septotemporal distribution and quantification of GAD67 mRNA expressing neurons at 2 and 21 days after KA injection.** *In situ* hybridization for GAD67 mRNA was performed as described in Section “Materials and Methods.’ Representative coronal sections of four septotemporal levels are shown. **(A–D) **Control mouse, septal **(A, B**), intermediate **(C)** and temporal **(D)** hippocampus. GAD67 mRNA-expressing cells were distributed throughout the hippocampus but accumulated close to the granule cell layer and pyramidal cell layer. This pattern was comparable at all septotemporal levels. Granule cells in the dentate gyrus show weak GAD67 mRNA expression. **(E–H)** Contralateral hippocampus, 2 days after KA injection, septal **(E, F)**, intermediate **(G)** and temporal level **(H)**. The distribution and density of GAD67 mRNA-positive cells was comparable to controls at all septotemporal levels. In some mice GAD67 mRNA-positive cells were slightly reduced in distal CA1 of the septal hippocampus **(E)**. GAD67 mRNA expression in granule cells was comparable to controls or slightly upregulated in the septal hippocampus only. **(I–L)** Ipsilateral hippocampus, 2 days after KA injection. **(I, J)** In the septal hippocampus most GAD67 mRNA-expressing cells were lost except for a group of cells in CA3/CA2 (arrow). GAD67 mRNA expression in granule cells was upregulated. **(K)** In the more dorsal part of the intermediate hippocampus GAD67 mRNA-expressing interneurons were lost, whereas in the more ventral parts they were preserved. GAD67 mRNA expression in granule cells was upregulated. **(L) **In the temporal hippocampus GAD67 mRNA-expressing interneurons were only lost in the very dorsal parts of the section but preserved elsewhere, GAD67 mRNA expression in granule cells was comparable to controls. **(M–P)** Ipsilateral hippocampus, 21 days after KA injection. **(M, N)** In the septal hippocampus only a few GAD67 mRNA-expressing cells were preserved. Note that cells in CA3, which were present earlier, were lost at the late time point indicating a progressive interneuron loss. The apparent reduction of GAD67 mRNA upregulation in granule cells is most likely due to their reduced density owing to the prominent dispersion of the granule cell layer. **(O) **In the intermediate hippocampus GAD67 mRNA-expressing cells were lost to a larger extent than at 2 days after KA and GAD67 mRNA expression in the granule cells was still strongly increased. **(P)** In the temporal hippocampus, GAD67 mRNA-expressing interneurons were lost in the dorsal part of the section indicating the progressive loss of interneurons. GAD67 mRNA was upregulated only in dispersed granule cells (arrow marks the transition zone). In the ventral parts of the section GAD67 mRNA expression was comparable to controls. **(Q)** Quantification of GAD67 mRNA-expressing cells at four positions along the septotemporal axis in controls and at 2 and 21 days after KA. Cells were counted in the whole hippocampus and cell density is given as GAD67-positive cells/mm^2^(*n* = 5 each group). Values are displayed as mean ± SEM, statistical comparison was made with an ANOVA and Tukey’s multiple comparison test, significance values were set as follows: *p < 0.05, **p < 0.01, ***p < 0.001. **(R)** As in **(Q)**, but only the hilus was marked as region of interest. Note that differences at position three are only significant when only the area of the hilus is considered. **(S)** Enlargement of the transition zone, see box marked in **(P)**. A few GAD67 mRNA-positive cells appeared dorsal to the transition zone (arrows) but the full density of GAD67 mRNA-positive cells was only restored ventral to this area. Note that GAD67 mRNA upregulation affected only dispersed granule cells. **(T)** Quantification of GAD67 mRNA-expressing cells only in the areas of the hippocampus where GCD was present at 21 days (in contrast to **(Q)**, where the whole hippocampus was considered). The loss was only significant at 21 days, indicating the progressive loss of interneurons in the temporal hippocampus (*p* = 0.015, ANOVA, *p* < 0.05, Tukey’s post-test, *n* = 5 each group). **(U)** Same as in T but only for the area of the hilus (*p* = 0.008, ANOVA, *p* < 0.01, Tukey’s post-test, *n* = 5 each group). Scale bars: 200 μm, **S**: 50 μm. GCL, granule cell layer; H, hilus; CA1, CA2, CA3, *cornu ammonis*.

In control mice GAD67 mRNA-expressing interneurons were distributed in all hippocampal areas but accumulated in or close to the pyramidal cell layer, the granule cell layer and in the hilus at all septotemporal positions (controls: *n* = 7 mice, **Figures 3A–D**). The increased density of GAD67 mRNA-positive cells in the temporal hippocampus (**Figure 3D**) matches previous studies analyzing the septotemporal interneuron distribution in healthy mice (Jinno et al., 1998), but quantification revealed that the density of positive cells did not differ significantly from more anterior levels (**Figures 3Q,R**). GAD67 mRNA was also weakly expressed in granule cells of control mice, as described earlier in mice and rats (Schwarzer and Sperk, 1995; Sloviter et al., 1996).

In the contralateral hippocampus of KA-injected mice the distribution of GAD67 mRNA-positive cells was comparable to controls at all septotemporal levels at 2 days (**Figures 3E–H**) and 21 days after KA (data not shown), except for a small reduction in density in distal CA1 of the septal hippocampus in some mice. Quantification revealed that the number of GAD67 mRNA-expressing cells was comparable to controls at all septotemporal levels (**Figures 3Q,R**). GAD67 mRNA expression in granule cells was comparable to controls or only slightly upregulated in the septal hippocampus (**Figures 3E,F**) but unchanged in the intermediate and temporal hippocampus (**Figures 3G,H**).

In contrast, already at 2 days after KA injection, GAD67 mRNA expression was almost completely lost in the ipsilateral septal hippocampus, except for a group of interneurons clustered in CA3a/CA2 and a few scattered cells in the dentate gyrus (KA 2 days: *n* = 7 mice; **Figures 3I,J**). Quantification of GAD67 mRNA-positive cell bodies revealed a significant reduction of cell density in the ipsilateral septal hippocampus compared to controls and to the contralateral hippocampus (*n* = 5 for each group; position 1: ANOVA: *p* = 0.024, pairwise Tukey’s post-test: ipsilateral-control *p* < 0.001, ipsilateral-contralateral *p* < 0.01; position 2: ANOVA: *p* < 0.0001, pairwise: ipsilateral-control *p* < 0.001, ipsilateral-contralateral *p* < 0.001; **Figure 3Q**). In the intermediate hippocampus GAD67 mRNA-expressing cells were only partially lost in the dorsal parts of the section but preserved in ventral parts (**Figure 3K**). In the temporal hippocampus they were mostly preserved at 2 days after KA injection (**Figure 3L**). No significant differences in cell density of GAD67 mRNA-positive cells were observed in the intermediate and temporal hippocampus when we considered the area of the whole hippocampus for quantification (position 3: ANOVA: *p* = 0.55; position 4: ANOVA: *p* = 0.32; **Figure 3Q**). When considering only the area of the hilus, we found a significant reduction of interneuron density compared to controls and to the contralateral side at the level of the septal and the intermediate hippocampus but not at temporal sites (*n* = 5 each group; position 1: ANOVA: *p* < 0.0001, pairwise Tukey’s post-test: ipsilateral-control and ipsilateral-contralateral *p* < 0.01; position 2: ANOVA: *p* = 0.0005, pairwise: ipsilateral-control and ipsilateral-contralateral *p* < 0.001, position 3: ANOVA: *p* = 0.0018, pairwise: ipsilateral-control and ipsilateral-contralateral *p* < 0.01; position 4: ANOVA: *p* = 0.036, pairwise: not significant; **Figure 3R**). Concomitant with the loss of interneurons, we observed a strong upregulation of GAD67 mRNA expression in ipsilateral granule cells at all positions except for the temporal hippocampus (**Figures 3I–L**), which is in agreement with studies in rats (Schwarzer and Sperk, 1995), but in contrast to Makiura et al. (1999) who showed a transient increase of GABA but not of GAD67.

In the chronic phase at 21 days after KA injection, when GCD and recurrent epileptic activity had fully developed, GAD67 mRNA expression was even further decreased: in the septal hippocampus a few GAD67 mRNA-expressing cells remained in the dentate gyrus but GAD67 mRNA-expressing interneurons in CA3a/CA2 were lost at this time point (KA 21 days: *n* = 5 mice, **Figures 3M,N**). The reduction of interneurons in the septal hippocampus was significant compared to the contralateral hippocampus and controls (position 1 and 2: pairwise Tukey’s post-test: ipsilateral-control and ipsilateral-contralateral *p* < 0.001) but the further reduction compared to 2 days after KA injection did not reach significance (**Figure 3Q**). At this late time point, cell loss progressed further toward the temporal hippocampus (**Figures 3O,P**), however, due to the large septotemporal extent of coronal slices at position 3 and 4, this was only significant in the intermediate hilus (position 3: pairwise Tukey’s post-test: ipsilateral-control and ipsilateral-contralateral *p* < 0.001; **Figures 3Q,R**). Notably, when comparing the septotemporal extent of GCD and interneuron loss, we observed that GAD67 mRNA-positive interneurons were mostly lost at sites where GCD was present and their full density was only established temporal to the transition zone from GCD to normal granule cell layer width (**Figures 3S–U**). The upregulation of GAD67 mRNA expression in granule cells was still visible at 21 days but interestingly only in dispersed granule cells (**Figure 3P**). Immunocytochemical analysis with an antibody against GAD65/67 confirmed the region-selective loss of GAD65/67-expressing interneurons and upregulation of GAD65/67 in granule cells in the septal and intermediate, but not temporal hippocampus on the protein level (data not shown). In addition, it revealed a strong upregulation of GAD65/67 expression in ipsilateral mossy fiber terminals in the hilus and CA3 at 2 days after KA and throughout the granule cell layer, molecular layer and CA3 at 21 days after KA, reflecting mossy fiber sprouting.

**FIGURE 4:**
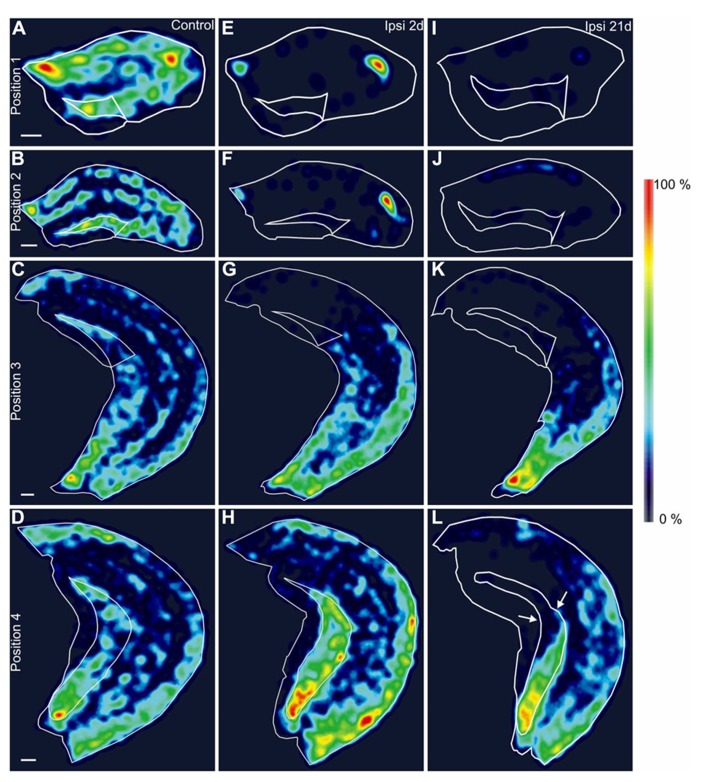
**Spatial distribution of GAD67 mRNA-positive interneurons.** **(A–L)** Density of GAD67 mRNA-expressing interneurons in representative hippocampal sections along the septotemporal axis displayed as heatmaps in false-color, relative to the maximal density within each position. The outer border of the hippocampus and the hilus are marked with white traces. Brighter color indicates higher, darker color lower relative cell density. **(A–D)** Control mouse, septal **(A, B)**, intermediate **(C)** and temporal hippocampus **(D)**. **(E–H)** Ipsilateral hippocampus, 2 days after KA injection, septal **(E, F)**, intermediate **(G)** and temporal level **(H)**. A gradient of cell loss along the septotemporal axis is visible. (**I–L)** Ipsilateral hippocampus, 21 days after KA injection, septal **(I, J)**, intermediate **(K)** and temporal level **(L)**. Note that the loss of GAD67 mRNA-expressing cells extends to further temporal areas than at 2 days after KA and the reduction of interneuron density can be observed beyond the transition zone from GCD to normal granule cell layer width (**L**, arrows). Scale bars: 200 μm.

For visualization of the distribution of GAD67 mRNA-expressing interneurons we generated heatmaps which display the cell density in false color (**Figure 4**). These maps illustrate the progressive loss of GAD67 mRNA expression between 2 and 21 days in the intermediate and temporal hippocampus (positions 3 and 4, **Figures 4G,H,K,L**). Furthermore, they show that reduced interneuron density extended to more temporal sites than GCD (**Figures 4K,L**), indicating the high sensitivity of GABAergic interneurons to KA injection and SE.

### PARVALBUMIN-POSITIVE INTERNEURONS ARE REDUCED BEYOND THE TRANSITION ZONE

**FIGURE 5:**
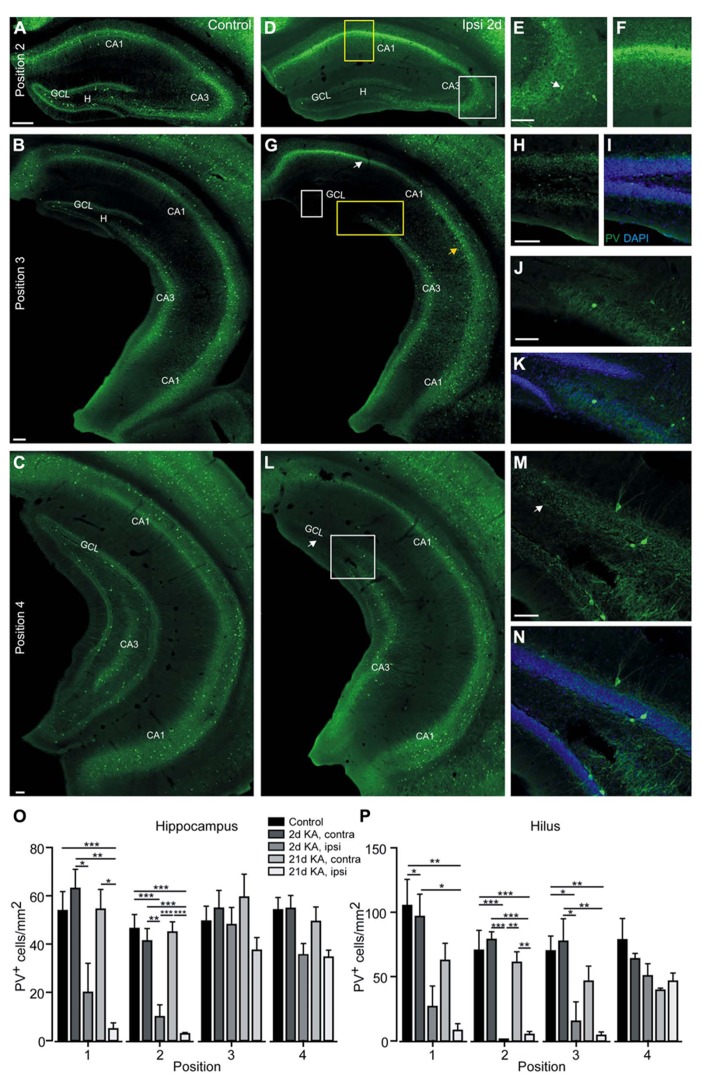
**Parvalbumin-positive cells are lost in the septal and intermediate hippocampus at 2 days after KA injection.** **(A–N)** Representative sections of an immunocytochemical staining for PV. **(A–C)** Control mouse, septal **(A)**, intermediate **(B)**, and temporal hippocampus **(C)**. PV-positive cells were mainly located in or close to the granule cell layer or the pyramidal cell layer and in *stratum oriens* and were equally distributed along the septotemporal axis. The PV-positive axon plexus surrounding the principal cell layers is visible. **(D–N)** Ipsilateral hippocampus, 2 days after KA. **(D–F)** In the septal hippocampus most PV-positive cells were lost, except for a few cells in CA3 (white box, enlarged in **E**, arrow marks PV-positive cell), and the PV-positive axon plexus was reduced particularly in the dentate gyrus and disorganized in CA1 (yellow box, enlarged in **F**). **(G)** The more dorsal areas of the intermediate hippocampus showed a cell loss pattern in CA1 comparable to the septal hippocampus (white arrow) whereas in the more ventral areas PV-positive cells were preserved (yellow arrow). In the dorsal areas of the dentate gyrus residual PV expression was observed close to the granule cell layer (white box, enlarged in **H** and **I**; with DAPI to highlight the granule cell layer). In the ventral areas of the intermediate dentate gyrus and in CA3 PV-expressing cells were preserved (yellow box, enlarged in **J** and **K** with DAPI). **(L)** In the temporal hippocampus PV-expressing cells were only reduced in the very dorsal parts of the section (arrow) but preserved elsewhere (white box, enlarged in **M** and **N** with DAPI). **(O)** Quantification of PV-positive interneurons at four positions along the septotemporal axis in controls and at 2 and 21 days after KA. Cells were counted in the whole hippocampus and cell density is given as PV-positive cells/mm^2^. Values are displayed as mean ± SEM (*n* = 6 controls, *n* = 4 KA 2 days, *n* = 5 KA 21 days), statistical comparison was made with an ANOVA and Tukey’s multiple comparison test, significance values were set as follows: *p < 0.05, **p < 0.01, ***p < 0.001. **(P)** As in **(O)**, but only the hilus was marked as region of interest. Note that at position two PV-positive cells in the hilus were nearly completely lost after KA injection. Scale bars: **A–D**, **G**, **L**, 200 μm; **E**, **F**, **H–K**, **M**, **N**, 100 μm.

To more specifically characterize the loss of interneurons along the septotemporal axis, we immunocytochemically stained PV-expressing interneurons since they are highly vulnerable in KA-injected mice surrounding the injection site (Bouilleret et al., 2000a). In controls, the majority of PV-expressing cells were located close to or within the principal cell layers (controls: *n* = 6 mice, **Figure 5A**), representing most likely axo-axonic and basket cells. A few PV-positive interneurons were also located in the hilus and *strata radiatum* and *oriens* of the CA region. The distribution of PV-positive cells was comparable at all septotemporal sites (**Figures 5A–C**), which was confirmed by quantification (**Figures 5O,P**) and matched previous results (Kosaka et al., 1987; Somogyi and Klausberger, 2005). In the contralateral hippocampus of KA-injected mice the density of PV-expressing cells was comparable to controls at all sites (**Figures 5O,P**). Due to the high similarity of positions 1 and 2, as shown for GAD67 mRNA expression, we only display positions 2–4 in the following photomicrographs.

Already at 2 days after KA injection PV-positive cell bodies were strongly reduced in the septal hippocampus except for a few cells in CA3 and distal CA1 (KA 2 days: *n* = 4 mice, **Figures 5D,E**). The PV-positive axon plexus in CA1 and in the inner molecular layer was also vanishing (**Figures 5D,F**). The strong fluorescence in the pyramidal cell layer of CA1 is comparable to what can be observed for many different antibodies and is most likely due to unspecific staining of degenerating cells. Quantification of PV-expressing interneurons revealed that the loss of these cells was significant compared to controls and the contralateral hippocampus for the hilus (*n* = 6 controls, *n* = 4 KA-injected mice; position 1: ANOVA: *p* = 0.0011, pairwise Tukey’s post-test: ipsilateral-control *p* < 0.05; position 2: ANOVA: *p* < 0.0001, pairwise: ipsilateral-control and ipsilateral-contralateral *p* < 0.001) as well as the whole hippocampus (position 1: ANOVA: *p* = 0.0002, pairwise: ipsilateral-contralateral *p* < 0.05; position 2: ANOVA: *p* < 0.0001, pairwise: ipsilateral-control *p* < 0.001 and ipsilateral-contralateral *p* < 0.01; **Figures 5O,P**).

In the intermediate hippocampus a characteristic pattern was observable: In the dorsal part the reduction of PV-positive cell bodies and partial preservation of the PV-positive plexus was comparable to the septal hippocampus (**Figures 5G–I**), while in the ventral part PV-positive cell bodies and their axon plexus seemed preserved (**Figures 5G,J,K**). This was confirmed by quantification which revealed a significant loss of PV-expressing cells in the hilus (position 3: ANOVA: *p* = 0.0013; pairwise: ipsilateral-contralateral and ipsilateral-contralateral *p* < 0.05) but no changes when the whole hippocampus was regarded (ANOVA: *p* = 0.30; **Figures 5O,P**). In the temporal hippocampus PV-positive cells and axons were only lost in the very dorsal parts of the section but preserved elsewhere (**Figures 5L–N**) and quantification showed comparable densities for the hilus (position 4: ANOVA: *p* = 0.10) and hippocampus (ANOVA: *p* = 0.02, pairwise: not significant; **Figures 5O,P**).

**FIGURE 6:**
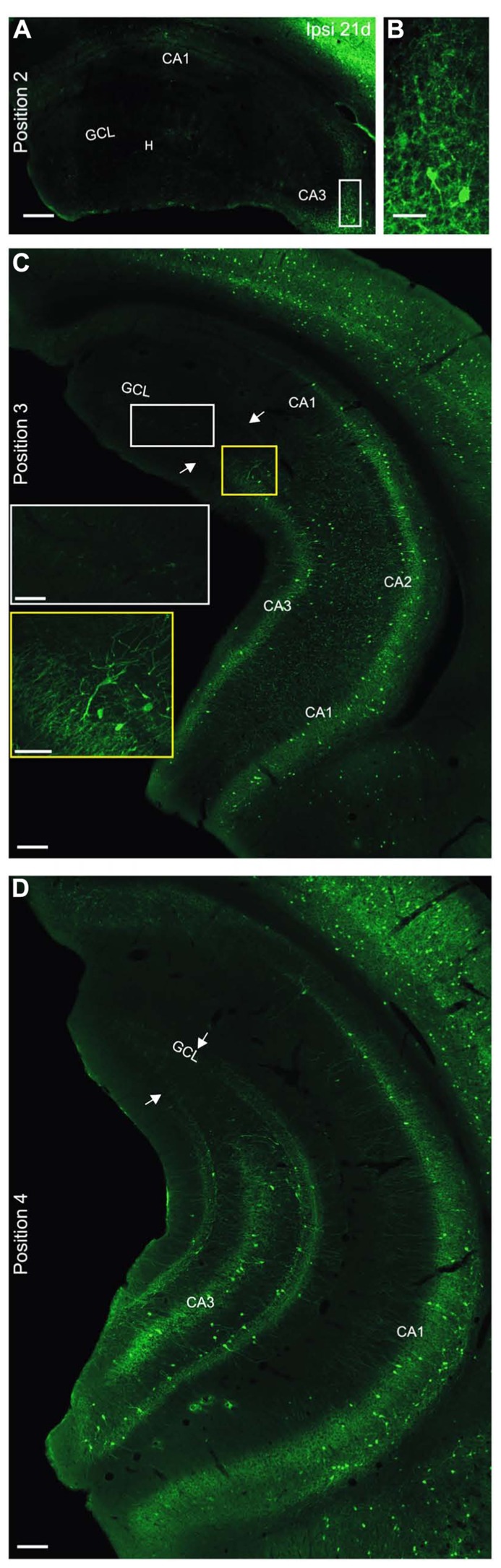
**Progressive loss of parvalbumin-positive cells at 21 days after KA injection. (A–D)** Ipsilateral hippocampus, 21 days after KA. **(A)** In the septal hippocampus not only the PV-positive cells but also the PV-positive axon plexus almost completely vanished, indicating a progressive loss. A few dysmorphic cells remained in CA3 in some slices (white box, enlarged in **B**). Note the prominent GCD. **(C)** In the intermediate hippocampus PV-positive cells and the PV-positive axon plexus were lost in the dorsal part (white box, enlarged in inset with white frame) but preserved at more ventral sites (yellow box, enlarged in inset with yellow frame). The loss extended to more ventral areas than at 2 days after KA. The transition zone from GCD to normal granule cell layer width is marked (arrows). Note that the loss of PV-positive cells extends slightly beyond this zone. **(D)** In the temporal hippocampus PV-expressing cells and the PV-positive plexus were gone in the dorsal parts but preserved elsewhere. The transition zone is marked with arrows. Scale bars: **A, C, D**, 200 µm; **B**, insets in **C**, 50 µm.

At 21 days after KA, PV-expressing cells in the septal hippocampus were nearly completely lost except for a very small group of dysmorphic neurons in CA3 (**Figures 6A,B**); furthermore, the PV-stained axon plexus in CA1 and the dentate gyrus was no longer visible (KA 21 days: *n* = 5 mice, **Figure 6A**). This reduction was significant when regarding the whole hippocampus (position 1: pairwise Tukey’s post-test: ipsilateral-control and ipsilateral-contralateral *p* < 0.01; position 2: pairwise: ipsilateral-control and ipsilateral-contralateral *p* < 0.001; **Figure 5O**) as well as the hilus (position 1: pairwise: ipsilateral-control *p* < 0.01; position 2: pairwise: ipsilateral-control *p* < 0.001, ipsilateral-contralateral *p* < 0.01; **Figure 5P**). In the intermediate hippocampus the loss of PV-positive cells and axons also progressed to slightly more ventral areas (i.e., toward the temporal hippocampus) than at 2 days after KA injection (**Figure 6C**). Yet, this difference was only significant for the hilus (position 3: pairwise: ipsilateral-control *p* < 0.01) but not for the whole hippocampus (**Figures 5O,P**). At the most temporal site (position 4), the progression of the loss of PV labeling between 2 and 21 days after KA was also apparent, in particular in the hilus (**Figure 6D**), however, this reduction was not significant (**Figure 5P**). Notably, PV-positive cell bodies were preserved only at sites temporal to the transition zone from GCD to normal granule cell layer width (**Figures 6C,D**).

### REDUCTION OF NPY-POSITIVE INTERNEURONS, BUT LOCATION-DEPENDENT UPREGULATION OF NPY IN MOSSY FIBERS AND GRANULE CELLS

**FIGURE 7:**
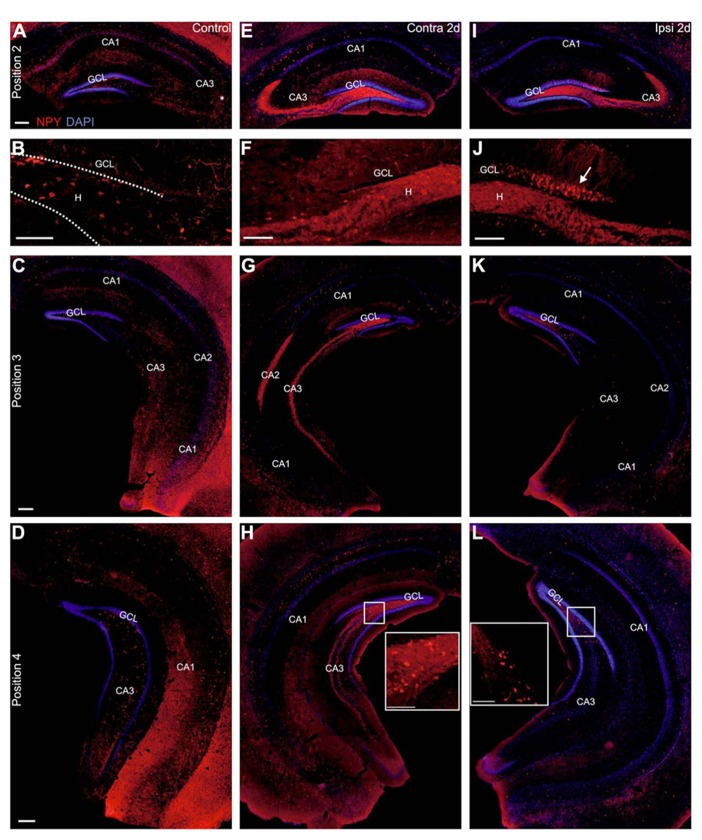
**Ectopic pattern of NPY staining at 2 days after KA injection.** **(A–L)** Representative sections of immunocytochemical staining for NPY (red) with DAPI counterstaining (blue) at 2 days after KA injection. **(A–D)** Control mouse, septal **(A, B)**, intermediate **(C)** and temporal hippocampus **(D)**. NPY-expressing cells were located mainly in the hilus and in *stratum oriens *and their connections were visible throughout the hippocampus. **(B)** Cutout of **(A)** with hilus and granule cell layer enlarged. NPY-positive cells were mostly located in the hilus and their axons were visible throughout the dentate gyrus but granule cells and mossy fibers were not stained. **(E–H)** Contralateral hippocampus. **(E)** In the septal hippocampus an upregulation of NPY expression in interneurons was visible in the CA region in *strata oriens*, *pyramidale* and *radiatum*. In addition, NPY was strongly upregulated in the mossy fibers. NPY-positive interneurons in the hilus were preserved but mostly outshined by the strong mossy fiber staining in the whole hilus (see enlarged in **F**). **(G, H)** In the intermediate and temporal hippocampus staining of NPY-positive interneurons was slightly upregulated in the dorsal part of the section but comparable to controls in the ventral part. NPY expression in the mossy fibers was strongly increased in the hilus, CA3 and CA2 at all septotemporal levels. **(I–L)** Ipsilateral hippocampus. **(I)** In the septal hippocampus NPY-expressing interneurons were lost except for a few remaining, strongly stained cells in CA3 *stratum oriens*. NPY expression was upregulated in the mossy fibers. In contrast to the contralateral side, NPY imunostaining was strongly enhanced in a large portion of granule cell bodies and dendrites (see enlarged in **J**, arrow). **(K)** In the intermediate hippocampus NPY-expressing cells were preserved in the hilus and expression in the CA region was comparable to controls. Upregulation of NPY in the mossy fibers was only visible in the very dorsal parts of the section. **(L)** In the temporal hippocampus NPY expression was comparable to controls. Scale bars: 200 μm; **B**, **F**, **J**, insets 100 μm.

In controls, NPY-positive cells were abundant in the hilus and many NPY-expressing cell bodies were visible in *stratum oriens* of CA3 and CA1 at all septotemporal levels (controls: *n* = 4 mice, **Figures 7A–D**), in line with previous reports in mice (Makiura et al., 1999) and rats (Deller and Leranth, 1990; Sperk et al., 2007; Kuruba et al., 2011). Some NPY-positive cells were present in *strata radiatum* and *pyramidale* of CA3 and CA1. In addition, a weakly stained NPY-positive axon plexus was visible throughout the hippocampus (**Figures 7A–D**).

At 2 days after KA injection, the contralateral hippocampus showed considerably enhanced NPY staining in cell bodies mainly in *strata oriens*, *pyramidale* and *radiatum* of CA1 of the septal hippocampus (*n* = 5 of 5 mice, **Figures 7E,F**) and in dorsally located parts of the intermediate and temporal hippocampus (4/5 mice, **Figures 7G,H**). In addition, NPY was slightly (2/5 mice) or even strongly upregulated in contralateral mossy fibers at all septotemporal levels (3/5 mice, **Figures 6E–H**). This upregulation spatially overlapped with NPY-positive interneurons and did not allow reliable quantification of NPY-positive cells. NPY expression in granule cells was comparable to controls.

In contrast, in the ipsilateral hippocampus, NPY-positive interneurons were lost in the septal hilus and strongly reduced in CA1, but mostly preserved in CA3 (KA 2 days: 5/5 mice, **Figures 7I,J**). In the intermediate and temporal hippocampus NPY-positive interneurons were preserved in the hilus and CA region (**Figures 7K,L**). More conspicuous, however, was the strong upregulation of NPY in septal granule cells and in dorsally located granule cells of the intermediate hippocampus and in their dendrites at 2 days after KA injection (5/5 mice, **Figures 7I–K**). In addition, mossy fibers showed a strongly increased NPY staining in the septal hippocampus (5/5 mice, **Figures 7I,J**), which extended to the intermediate and temporal hippocampus (3/5 mice) or vanished in ventral parts of the intermediate and temporal hippocampus (2/5 mice, **Figures 7K,L**).

**FIGURE 8:**
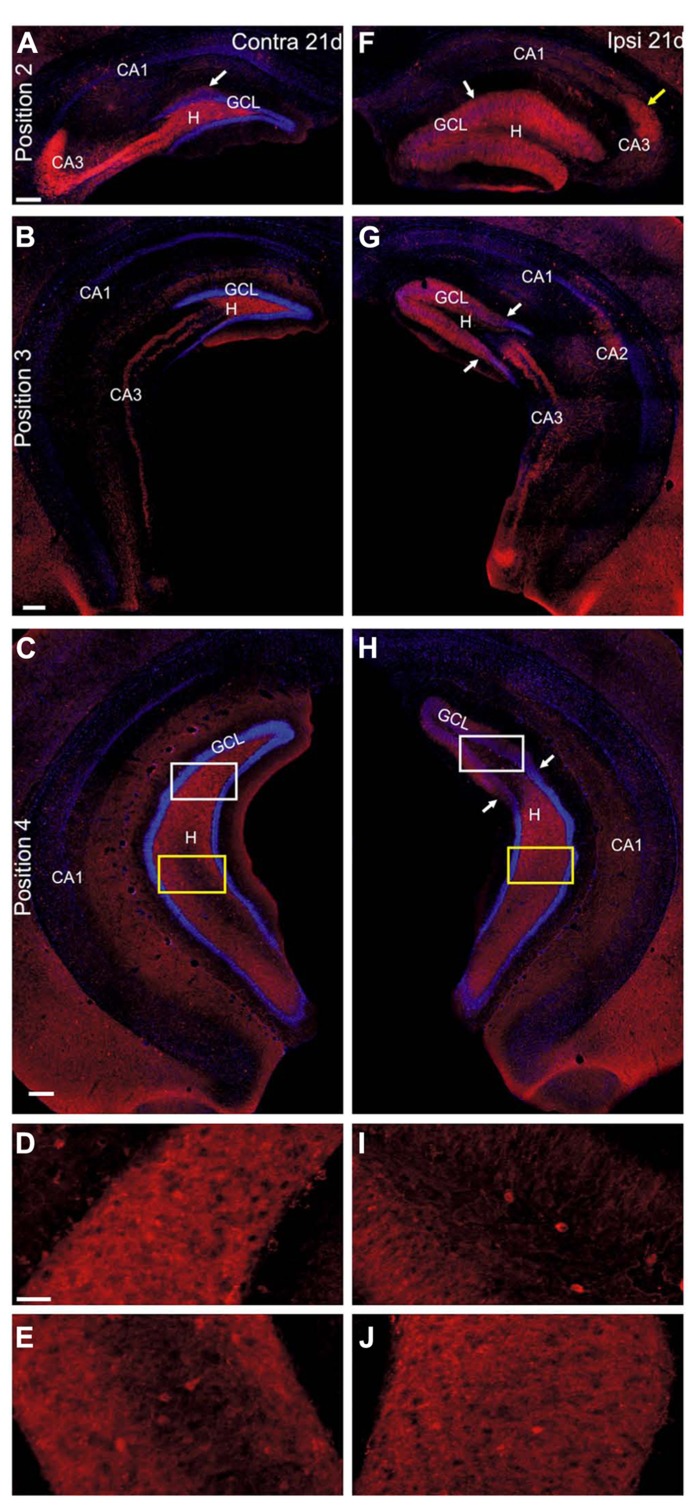
**NPY staining pattern changes between 2 and 21 days after KA injection.** **(A–J)** Representative sections of an immunocytochemical staining for NPY (red) with DAPI counterstaining (blue) at 21 days after KA injection. **(A–E)** Contralateral hippocampus, septal **(A)**, intermediate **(B)** and temporal hippocampus **(C–E)**. Upregulation of NPY in the CA region was no longer visible but NPY-expressing interneurons were distributed comparable to controls. NPY was upregulated in the mossy fibers at all septotemporal levels (white and yellow box in **C**, enlarged in **D** and **E**, respectively). Some terminals of NPY-positive fibers were seen in the inner molecular layer of the septal hippocampus (arrow in **A**). **(F–J)** Ipsilateral hippocampus. **(F)** In the septal hippocampus most NPY-positive interneurons were lost.

At 21 days after KA, the upregulation of NPY expression in interneurons in the contralateral hippocampus was no longer visible and staining of interneurons was comparable to controls in all mice (*n* = 6, **Figures 8A–E**). In contrast, the enhanced NPY labeling in mossy fibers was maintained at all septotemporal positions (**Figures 8A–E**), however, the intensity of labeling was variable across mice (3/6 mice with weak NPY upregulation, 3/6 mice with strong NPY upregulation) in agreement with Arabadzisz et al. (2005). In addition, in the septal hippocampus NPY-positive terminals were apparent in the inner molecular layer close to the granule cells (**Figure 8A**).

Instead, NPY was strongly upregulated in mossy fibers. Note that mossy fibers had sprouted into the dispersed granule cell layer and molecular layer (white arrow). In addition a termination zone in distal CA3/CA2 was visible (yellow arrow). In contrast to the earlier time point, granule cell bodies were not strongly NPY-positive. **(G)** In the intermediate hippocampus NPY-positive interneurons were preserved in the ventral parts of the section, however, starting from areas located more septal than the transition zone from GCD to normal granule cell layer width (arrows). In the dorsal part of the section a pattern of mossy fiber sprouting comparable to the septal hippocampus occurred whereas in the ventral parts NPY was still upregulated in mossy fibers but at their normal position. Note the overlap of sprouted and normal mossy fibers. **(H)** In the temporal hippocampus NPY-positive interneurons were preserved slightly dorsal to the transition zone (arrows), where GCD was still visible and mossy fiber sprouting occurred (white box, enlarged in **I**). Preservation of NPY-positive cells was also seen more ventrally where enhanced NPY expression was seen in mossy fibers in the hilus (yellow box, enlarged in **J**). Scale bars: 200 μm; **D**, **E**, **I**, **J**, 50 μm.

In the ipsilateral septal hippocampus the progressive loss of NPY-positive cells was more pronounced than at 2 days after KA and also affected CA3 (KA 21 days: *n* = 6 mice, **Figure 8F**). In the ipsilateral intermediate and temporal hippocampus NPY expression in cell bodies was preserved in the hilus and CA region in all mice (**Figures 8G,H**). Remarkably, NPY-positive cell bodies were visible in the hilus at sites where GCD was present (i.e., septal to the transition zone, **Figures 8H,I**), which is in contrast to PV. At 21 days the staining of mossy fibers has substantially changed compared to 2 days: in the septal hippocampus NPY-labeled mossy fibers were observed throughout the granule cell layer and molecular layer and at a small spot in CA3/CA2 (**Figures 8F–H**), resembling the spatial pattern of Timm staining (Bouilleret et al., 1999) and thus most likely reflects mossy fiber sprouting. This pattern was present at all positions where GCD occurred and extended even slightly beyond the transition zone (**Figures 8G,H**). Toward the temporal pole NPY labeling in mossy fibers was enhanced in the hilus and CA3, indicating that mossy fiber sprouting and normal mossy fiber localization overlapped (*n* = 6, **Figures 8H,J**).

## DISCUSSION

In the current study we compared the intrahippocampal spread of KA-induced SE with the spatial pattern of interneuron loss along the septotemporal axis of the hippocampus. We show that in the ipsilateral septal hippocampus most GAD67 mRNA-expressing interneurons were lost already at 2 days after KA injection except for a small population of interneurons in CA3 including PV- and NPY-positive cells. Cell loss extended to the intermediate hippocampus already at 2 days, but progressed further toward the intermediate hippocampus and to CA3 until 21 days. In the intermediate hippocampus we observed a differential vulnerability of the different interneuron populations: PV-expressing cells were lost at much more temporally located areas than NPY-expressing cells. In addition to the loss of interneurons, we show that GAD67 mRNA was exclusively upregulated in dispersed granule cells, whereas upregulation of NPY expression occurred in ipsilateral septal granule cells and in mossy fibers throughout the hippocampus, most likely representing a compensatory mechanism to the loss of inhibition.

### INTERNEURONS WERE LOST BEYOND THE SCLEROTIC AREAS

In our previous study, we demonstrated that recurrent epileptiform activity is not strongest in the most sclerotic areas of the septal hippocampus, but instead in the intermediate hippocampus, which shows only minor histological changes (Häussler et al., 2012). Here, we thus analyzed how inhibition could contribute to differential strength of epileptiform activity by mapping the distribution of interneurons along the entire septotemporal axis. To this end, we quantified the distribution of GAD67 mRNA-expressing cells and PV-expressing interneurons along the septotemporal axis of the hippocampus. In our quantification PV-positive interneurons make up for 8–13% of the total density of GAD67-positive interneurons in the hilus and 12–15% in the whole hippocampus which is lower than what has been shown in the literature (14–20% in the hilus, 20–24% in the hippocampus; Freund and Buzsaki, 1996). A higher yield and better signal-to-noise ratio in *in situ* hybridization for GAD67 mRNA compared to immunocytochemistry for PV expression might lead to this underestimation of the relation. However, as the relation is comparable for controls and KA-injected mice for all four positions, it does not influence our results on changes in the distribution of PV-positive cells in TLE.

In addition to the nearly complete loss of GAD67 mRNA-expressing interneurons in the septal hippocampus at 21 days after KA injection, which has been previously shown (Bouilleret et al., 2000a,b), our analysis revealed that the loss of interneurons is not restricted to the sclerotic area, but extends to the intermediate hippocampus. More precisely, the loss of interneurons extends even beyond the transition zone from GCD to normal granule cell layer width. In particular interneurons expressing PV showed a high vulnerability: PV-positive somata, as well as the dense PV-positive axon plexus were also only visible at sites temporal to this transition zone.

We are aware that the loss of PV expression might not be equivalent to the death of these interneurons and might reflect only a transient down-regulation of PV synthesis as suggested previously (Sloviter et al., 1991; Scotti et al., 1997; Wittner et al., 2001), or a transient reduction of inhibitiory function followed by structural or functional reconstruction, as shown in other epilepsy models (Houser and Esclapez, 1996; Hellier et al., 1999; Bernard et al., 2000; Holtkamp et al., 2005; Sloviter et al., 2006). However, given the loss of GAD67 mRNA expression at the same sites and neuronal death mainly in the hilus, close to the granule cell layer and in the CA region, as shown by Fluoro-Jade B staining, it is highly likely that the loss of PV expression reflects the death of these inhibitory interneurons instead of a transient loss of function. This is supported by a recent study in which granule cell responses upon perforant path stimulation were recorded *in vivo* in intrahippocampally KA-injected mice: granule cells showed oscillatory responses and reduced paired pulse inhibition already at 3 days after KA and the number of population spikes was even increased at 21 days after KA while paired pulse inhibition did not recover (Rougier et al., 2005), indicating the loss of inhibitory neurons.

Conflicting results on vulnerability of PV-expressing interneurons in human TLE and animal models exist in the literature (de Lanerolle et al., 1989; Sloviter et al., 1991; Zhu et al., 1997; van Vliet et al., 2004; Andrioli et al., 2007; Wyeth et al., 2010; Kuruba et al., 2011). Our data show that the almost complete loss of PV-positive interneurons explains only partially the total loss of GAD67 mRNA-positive interneurons and preliminary analyses revealed that this difference is not fully explained by the loss of NPY-expressing interneurons. This is in agreement with previous studies, in which, additionally, the loss of somatostatin-positive interneurons (Bouilleret et al., 2000b; Dugladze et al., 2007), calretinin- and calbindin-positive interneurons has been shown in the septal hippocampus of KA-injected mice (Bouilleret et al., 2000a). Mapping the contribution of these additional interneuron subtypes to total interneuron loss along the septotemporal axis is a goal for future studies.

The nearly complete loss of interneurons in the septal hippocampus in our model might be due to the strong sclerosis in this area and may represent an overestimation of what happens in human TLE. However, a correlation between the loss of pyramidal cells and the loss of interneurons in the hilus and in CA1 has also been shown in human TLE (Thom et al., 2012). In addition, that study showed that gradients of cell loss of principal cells and interneurons occur along the human longitudinal hippocampal axis in TLE with a trend toward stronger loss in the anterior and intermediate hippocampus. These data strongly support the transferability of our animal model to the human pathology. Furthermore, the expansion of cell loss toward the intermediate hippocampus and toward CA3 between 2 and 21 days in our study indicates that interneuron loss cannot be completely due to excitotoxicity of KA but that also SE and recurrent epileptiform activity have a destructive effect on interneurons (Mello and Covolan, 1996) and lead to progression of the disease as suggested by others (van Vliet et al., 2004).

Since the lack of PV-expression has been associated with increased seizure susceptibility (Schwaller et al., 2004), it seems likely that the strong reduction of perisomatically inhibiting PV-positive interneurons in the septal and intermediate hippocampus indeed contributes to the increased epileptogenicity in these regions. In the septal hippocampus, where dispersed granule cells show diminished excitability (Young et al., 2009), this might result in weaker epileptiform activity than at sites adjacent to the transition zone, matching the results of our previous study (Häussler et al., 2012). Yet, hypothesizing such an effect only from our current results is difficult due to the complex function of perisomatic and dendritic inhibition in the dentate gyrus since it actually might have a depolarizing effect due to the low resting potential of granule cells (Sauer et al., 2012). Our data thus highlight the importance of studying in detail the interaction between granule cells and interneurons in the structurally preserved intermediate hippocampus.

### DIFFERENTIAL UPREGULATION OF NPY AND GAD67 EXPRESSION

The loss of NPY-expressing interneurons in the ipsilateral septal hippocampus at 2 days after KA, the slight progress of cell loss until 21 days, as well as the transient upregulation of NPY in septal granule cells only at 2 days are in agreement with previous results (Makiura et al., 1999). However, the upregulation of NPY in interneurons in the contralateral CA1 region at 2 days after KA has not been described in this model previously. The differing pattern of NPY expression in the ipsi- and contralateral hippocampus in particular at 2 days suggests that it is driven by differential activation during SE. In fact, we found that SE activity was strong in the contralateral septal hippocampus but weak in the ipsilateral septal hippocampus, most likely due to a depolarization block in granule cells close to the injection site (Le Duigou et al., 2005). Accordingly, systemic KA injection in rats has shown that strong convulsions cause upregulation of NPY in granule cells while slight convulsions induce NPY upregulation in interneurons (Gruber et al., 1994). Furthermore, several studies demonstrated that high doses of glutamate agonists injected into one hippocampus provoke upregulation of NPY in contralateral interneurons (Gruber et al., 1994; Schwarzer and Sperk, 1998), whereas subneurodegenerative doses cause upregulation of NPY only ipsilaterally. Factors regulating NPY expression include interleukins (Barnea et al., 2001) or BDNF (Nawa et al., 1994) and, indeed, the increased expression of BDNF observed at 2 days after intrahippocampal KA injection in mice was stronger in the ipsilateral than in the contralateral granule cell layer (Heinrich et al., 2011).

In addition to the short-time effect, characterized by a transient upregulation of NPY in ipsilateral granule cells and contralateral interneurons, we observed an additional long-term effect characterized by upregulation of NPY in ipsi- and contra-lateral mossy fibers. This is already visible at 2 days but augments in the following, which is salient in mossy fibers of the ipsilateral intermediate and temporal hippocampus showing strong NPY labeling only at the late time point. A comparable augmentation of NPY with time after the initial insult has also been observed in pilocarpine-injected mice (Borges et al., 2003). The long-term effect might either be induced by gene expression patterns different from short-term effects, or, more likely by epileptiform activity, since the so-called silent period after intrahippocampal KA injection is not fully silent but characterized by isolated high amplitude spikes or short groups of epileptiform spikes at all septotemporal levels of both hippocampi (Arabadzisz et al., 2005; Meier et al., 2007; Häussler et al., 2012).

Upregulation of NPY is considered as endogenous antiepileptic mechanism protecting the brain against seizure-related damage (Klapstein and Colmers, 1997; Vezzani et al., 2002). Our study shows that this compensatory effect is present on the whole septotemporal extent of both hippocampi, yet, the different patterns septal and temporal to the transition zone reflect different structural changes: in the septal hippocampus excitation is augmented by recurrent mossy fiber sprouting (Cronin et al., 1992) and this recurrent excitation is possibly attenuated by NPY in sprouted mossy fiber terminals. There is evidence in pilocarpine-injected mice that sprouted mossy fibers release NPY acting via the Y2 receptors which induce an inhibitory effect despite the recurrence (Nadler et al., 2007). In temporal areas and in the contralateral hippocampus CA3 pyramidal cells are preserved and since the intrinsic recurrent connectivity renders this region strongly excitable, mossy fiber input needs to be attenuated there. Interestingly, in the intermediate hippocampus both mechanisms are observable: mossy fibers still sprout into the molecular layer at sites with normal granule cell layer width but due to the preservation of CA3 the mossy fiber output to CA3 also seems to be compensated.

A second, possibly compensatory mechanism is the upregulation of GAD67 in granule cells which mainly occurs in the septal and intermediate ipsilateral hippocampus at 2 days after KA and is restricted to dispersed granule cells at 21 days after KA. This is in agreement with results in human TLE where strong upregulation of GAD67 has been shown in granule cells (Sperk et al., 2012) and with other animal models (Schwarzer and Sperk, 1995; Ramirez and Gutierrez, 2001). It has been shown that mossy fiber boutons release glutamate and GABA under healthy conditions (Beltrán and Gutiérrez, 2012) and that functional GABAergic transmission occurs in mossy fibers of hippocampal slices from rats after kindling (Gutierrez and Heinemann, 2001), suggesting that the upregulation of GAD67 in granule cells in our mice also leads to locally increased GABAergic transmission. To clarify whether this transmission has an effect compensating hyperexcitability requires experiments in slices from different septotemporal levels since the influence on CA3 pyramidal cells and, via mossy fiber sprouting-induced backpropagation, on other granule cells might be different.

### DOES THE INTERMEDIATE HIPPOCAMPUS CONSTITUTE AN EPILEPTOGENIC NETWORK?

Our study indicates that the intermediate hippocampus comprises multifaceted changes including the transition from GCD to normal granule cell layer width, differential vulnerability of PV- and NPY-expressing cells, and recovery of neurogenesis (Häussler et al., 2012). All these changes might be functionally linked. GCD is caused by migrating granule cells which lose their positional information through the loss and functional inactivation of Reelin (Haas et al., 2002; Heinrich et al., 2006; Müller et al., 2009; Tinnes et al., 2011) which normally acts as a position signal during development and in the adult. In the healthy hippocampus, Reelin is expressed by interneurons in the hilus, at the hippocampal fissure and in *stratum oriens* of the CA region (Pesold et al., 1998). In particular, the hilar interneurons are lost after KA injection as shown in our study. Interestingly, Reelin-expressing interneurons co-express NPY or somatostatin, but not PV (Pesold et al., 1999). The loss of PV-expressing interneurons in our study extended beyond GCD, but NPY-expressing cells were visible slightly more septally indicating that the extent of NPY cell loss might influence the septotemporal extent of GCD through expression gradients of Reelin. Another important fact is that NPY promotes hippocampal neurogenesis (Howell et al., 2003,^2005^; Decressac et al., 2011) and we previously showed differential regulation of neurogenesis with respect to strength of the initial SE: neurogenesis is lost in the septal hippocampus and only present in the intermediate and temporal hippocampus (Häussler et al., 2012). In fact, in the intermediate, temporal and contralateral hippocampus neurogenesis is strongly increased compared to controls which might be stimulated by the expression of NPY in non-sprouted mossy fibers together with the expression of NPY in surviving interneurons.

Does the transition zone have a functional role? In our previous study, we have shown that epileptiform activity was not strongest at sites where hippocampal sclerosis was most pronounced but, instead, in the adjacent area of the transition zone where neurogenesis reappeared. Neurogenesis, together with the reduction of inhibitory interneurons in this region, might comprise a network with disturbed excitation-inhibition balance and high epileptogenicity. The functional consequences of such gradual changes might be transferred to human TLE in which gradients of cell loss along the longitudinal hippocampal axis have been shown (Thom et al., 2012). In addition, our precise quantitative analysis of interneuron densities and neurogenesis in the whole hippocampus paves a way for modeling studies on the epileptogenicity of small shifts in network balance.

## Conflict of Interest Statement

The authors declare that the research was conducted in the absence of any commercial or financial relationships that could be construed as a potential conflict of interest.
